# A Microfluidic Device for Detecting the Deformability of Red Blood Cells

**DOI:** 10.3390/bios15110758

**Published:** 2025-11-14

**Authors:** Wenyan Liu, Liqiang Xie, Jiangcun Yang, Xiaobo Gong, Dan Sun, Ce Zhang

**Affiliations:** 1 State Key Laboratory of Photon-Technology in Western China Energy, Institute of Photonics and Photon-Technology, Northwest University, No. 1, Xuefu Avenue, Xi’an 710127, China; 2 School of Physics, Northwest University, No. 1, Xuefu Avenue, Xi’an 710127, China; 3 Department of Transfusion Medicine, Shaanxi Provincial People’s Hospital, Xi’an 710068, China; 4 School of Ocean and Civil Engineering, Shanghai Jiao Tong University, Shanghai 200240, China

**Keywords:** red blood cell deformability, microfluidic device, diabetes, single-cell analysis

## Abstract

Red blood cell (RBC) deformability is a critical biophysical property that enables effective passage of RBCs through microvasculature and ensures proper oxygen delivery. Impairment of this property is associated with various pathological conditions, including type 2 diabetes mellitus (T2DM). In this study, we developed an automated microfluidic platform for high-throughput and real-time assessment of RBC deformability under controlled flow conditions. The device features a structured microchannel design and integrated imaging to quantify individual cell deformation responses. Comparative analyses of RBCs from healthy individuals and T2DM patients revealed significant reductions in deformability in the diabetic group. In vivo validation using a diabetic mouse model further confirmed the progressive decline in RBC deformability under chronic hyperglycemia. This microfluidic approach provides a robust and efficient tool for characterizing RBC mechanical properties, offering potential for disease monitoring and clinical diagnostic applications.

## 1. Introduction

Red blood cells (RBCs) are highly specialized, deformable cells whose primary physiological role is to deliver oxygen from the lungs to peripheral tissues [1,2]. To fulfill this function, RBCs must traverse the narrowest capillaries—often with diameters smaller than the cells themselves—by undergoing substantial shape deformation without compromising membrane integrity [3,4]. This extraordinary deformability is governed by a delicate balance of cytoskeletal architecture [5,6,7], membrane composition [8,9], and intracellular viscosity [10,11], all of which can be altered by pathological states.

In various systemic diseases, such as diabetes mellitus [12,13,14], hypertension [15,16], sepsis [17,18,19,20], and sickle cell disease [21,22,23], the mechanical properties of RBCs are significantly impaired, leading to decreased microvascular perfusion [24,25], tissue hypoxia [26,27], and increased risk of vascular complications [28,29,30]. Among these, type 2 diabetes mellitus (T2DM) is particularly noteworthy due to its rising prevalence and its well-documented association with chronic hyperglycemia-induced damage to blood components [31,32,33]. Hyperglycemia can promote oxidative stress [34,35,36], membrane glycation [37,38], and cytoskeletal remodeling in RBCs [39,40], ultimately reducing their deformability. Decreased RBC flexibility contributes to elevated blood viscosity [41,42], increased shear stress in vessels [43], and impaired oxygen delivery [44,45], all of which play a role in diabetic microangiopathy.

Accurate and high-throughput assessment of RBC deformability is therefore essential not only for fundamental hematological studies [46] but also for early diagnosis [47], disease monitoring [48], and evaluation of treatment efficacy in patients with metabolic and vascular disorders [49]. Conventional techniques such as ektacytometry, micropipette aspiration, and optical tweezers have offered valuable insights into RBC mechanics, but they are often limited by low throughput, high operational complexity, and poor integration with clinical workflows [50].

In recent years, microfluidic technologies have emerged as powerful tools for biomechanical phenotyping of cells [51]. By mimicking physiological flow environments and allowing precise control of shear stress, microfluidic systems enable real-time, label-free, and high-throughput analysis of RBC deformation [52]. Moreover, microfluidics facilitates integration with imaging and automation modules, making it a promising approach for translational applications [53,54].

In this study, we present an automated microfluidic platform designed to evaluate RBC deformability with high temporal and spatial resolution. The system leverages a structured microchannel design to induce deformation and employs automated imaging to track cell morphology dynamically. We apply this platform to compare RBC deformability between healthy individuals and patients with T2DM and further validate its utility in a diabetic mouse model. Our findings support the potential of microfluidic deformability assessment as a functional biomarker for disease progression and therapeutic response in diabetes and related vascular conditions.

## 2. Materials and Methods

### 2.1. Fabrication of Microfluidic Chips

The chip design was created using AutoCAD 2018 and fabricated with standard UV lithography using SU-8 3005 photoresist (Microchem, Westborough, MA, USA). The SU-8 3005 was spin-coated at 8000 rpm to form rectangular fluid channels approximately 2 μm high. The chip was manufactured using soft lithography. In the process, 50 g of PDMS (monomer to catalyst ratio 10:1) was poured into the mold, degassed in a vacuum chamber for 1 h, and cured at 80 °C for 2 h. After curing, the PDMS chip was bonded to a glass substrate following plasma treatment and post-cured at 80 °C for over 8 h.

### 2.2. Chip Operation

After passing the pressure test and sterilization, the chip was connected to a syringe pump via fluidic tubing and managed using a MATLAB R2022b control program. The optimal closure pressure for the PDMS membrane valve was determined, with the chip operating within a typical pressure range of 25–30 psi. Before use, the channels were filled with PBS and degassed, followed by a 1 h incubation with 0.1% pluronic@F-68 solution to prevent cell adhesion, and then continuously rinsed with PBS.

### 2.3. Image Acquisition and Data Analysis

Fluorescence images were acquired using a Nikon Ti2-Eclipse microscope equipped with an automated stage and a digital CMOS camera (ORCA-Flash 4.0, Hamamatsu, Japan), with image acquisition controlled by Nikon’s NIS Elements AR 5.41.00 64-bit software. The remaining images were acquired using a custom integrated optical imaging system, equipped with a digital CMOS camera (ToupCam U3CMOS05100KPA; Hangzhou Tupu Optoelectronics Technology Co., Ltd., Hangzhou, Zhejiang, China), with image acquisition controlled by the Automated Liquid Control and Image Acquisition_1 software (custom-written in MATLAB R2022b), developed in MATLAB. Subsequently, image analysis was performed using the ImageXcel program (custom-written in MATLAB R2022b), also developed in MATLAB, following a three-step workflow: template construction, target recognition, and quantitative analysis. First, feature templates of microfluidic chips and RBCs were extracted from annotated GIF files via Canny edge detection and morphological dilation (disk-shaped structuring element, radius = 5 pixels). For target recognition in test images, a normalized cross-correlation (NCC) matching algorithm was applied, with a correlation threshold of 0.65 for chip recognition (fixed bounding box: 156 × 200 pixels) and 0.78 for RBC recognition. RBCs were further constrained by a minimum *Y*-axis height of 26 pixels, spatial localization within chip regions, and alignment with the chip’s central axis. Non-maximum suppression (minimum inter-object distance = 50 pixels) and overlap removal (based on intersection criteria) were used to eliminate redundant annotations.

For quantitative analysis, RBC centroids were localized via adaptive threshold segmentation (sensitivity = 0.5), and vertical distances from RBC centroids to the chip bottom were calculated (pixel-to-physical conversion factor = 0.33). Reliability was validated through visual inspection (display of green/red bounding boxes and centroids), single-RBC-per-chip constraints, and Gaussian distribution fitting (histogram with normality test), which collectively minimized false positives/negatives and ensured accuracy and stability of the results.

### 2.4. Mouse Model Development

A type 2 diabetes mellitus (T2DM) model was developed in C57BL/6J male mice via high-fat diet (HFD) and low-dose streptozotocin (STZ). Six-to-eight-week-old SPF C57BL/6J male mice (weighing 18–22 g) were divided into experimental and control groups (three mice each). The experimental group was fed an HFD (60% fat, 20% carbohydrates, 20% protein) for four weeks to induce obesity and insulin resistance, while the control group received a standard diet (10% fat). During this period, mouse weights were monitored weekly. Subsequently, the experimental group received intraperitoneal STZ injections (40 mg/kg/day in 0.1 mol/L citrate buffer, pH 4.5) for four consecutive days, with STZ prepared fresh daily and protected from light. The control group received equal volumes of citrate buffer. Successful model establishment was confirmed by measuring random blood glucose levels, with criteria being fasting glucose > 11.1 mmol/L or random glucose > 16.7 mmol/L.

### 2.5. Blood Sample Preparation

The study was approved by the Ethics Committee of Shaanxi Provincial People’s Hospital [(2020) R005]. 76 volunteers were recruited, after obtaining informed consent, approximately 20 μL of blood was collected from each volunteer via finger prick using a disposable lancet and collected in heparinized microtainers. The blood was diluted 20 times with PBS (pH 7.4) to 400 μL, centrifuged at 1000 rpm for 5 min within 4 h at 4 °C, and the supernatant was discarded. RBC were washed twice with PBS and finally resuspended in 400 μL of PBS to form a single-cell suspension for microfluidic chip experiments. For mouse blood collection, the tail vein method was used. C57BL/6J male mice (6–8 weeks old) had their tails wiped with 75% ethanol and soaked in warm water (37 °C) for 1 min to promote congestion. The tail tip was clipped with sterile scissors to collect 20 μL of blood into microtainers containing EDTA or heparin. After hemostasis with a cotton ball, the blood was diluted 20 times with PBS containing 1% BSA to 400 μL and incubated at 37 °C in a 5% CO_2_ incubator for 60 min. Post-incubation, the sample was centrifuged at 4 °C and 1000 rpm for 5 min, the supernatant was discarded, and the cells were resuspended in 400 μL of pre-cooled PBS (4 °C). This washing process was repeated twice to remove residual BSA and serum, and the cells were finally resuspended in 400 μL of PBS to form a homogeneous single-cell suspension for microfluidic chip analysis.

### 2.6. Calculation of RBC Surface Area and Volume

The surface area and volume of RBCs were calculated based on the method described in reference [55].

### 2.7. Statistical Analysis

All statistical analyses were performed using Student’s *t*-test as identified in Section 3. *p* < 0.05 value was considered statistically significant.

### 2.8. Numerical Simulation

Numerical simulations were performed using COMSOL Multiphysics^®^ 5.3. A 2D schematic of the chip, generated via CAD 2018 software, was imported into the simulation environment. The inlet flow velocity was set to 3 mm/s, a zero-pressure boundary condition was applied at the outlet, and a no-slip boundary condition was set for the microchannel walls. In the simulations, laminar flow physics was utilized to investigate transient results, and velocity data were collected at different positions using probes (see Supplementary Text for detailed parameters) [56,57,58,59,60,61,62,63,64,65,66].

## 3. Results

### 3.1. Design and Structure of the Microfluidic Device

**Figure 1:**
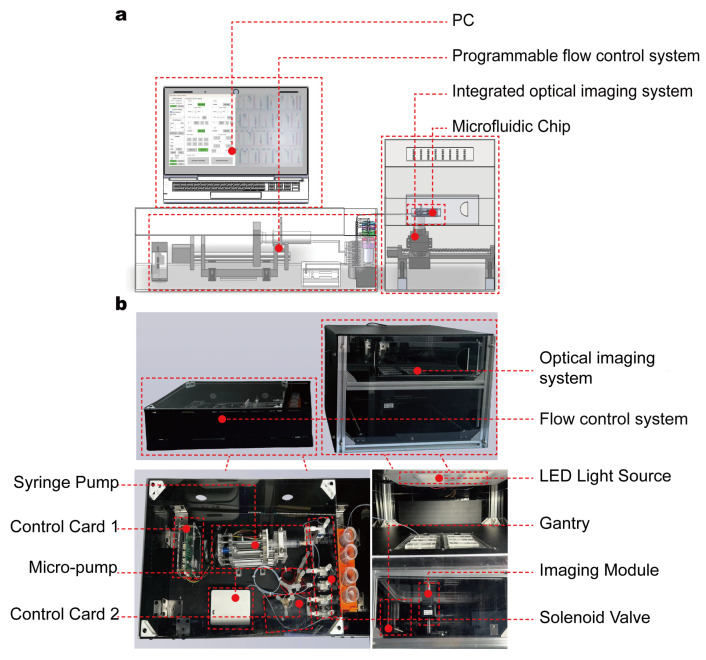
Automated microfluidic device for high-throughput, real-time evaluation of RBC deformability. (**a**) Schematic diagram of the device. (**b**) Photograph of the actual device.

**Figure 2:**
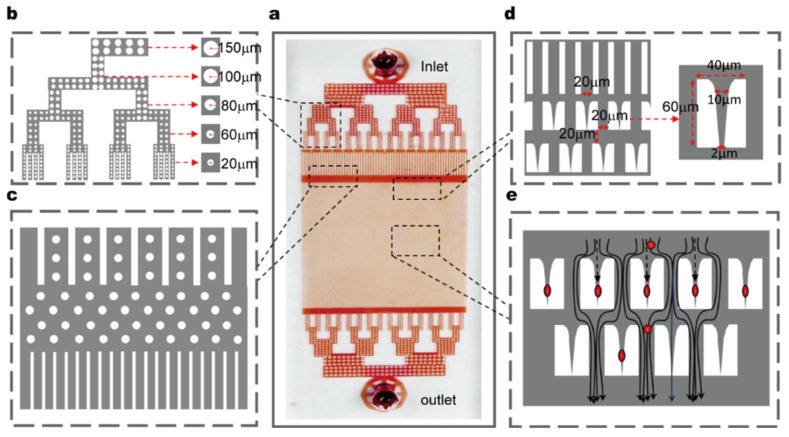
Design and structure of the microfluidic chip. (**a**) The microfluidic chip. (**b**) Detailed view of the dendritic branch channels at the inlet and the cylindrical pillars inside the channels. (**c**) Schematic diagram of the structure of the buffer zone and the array of straight channels. (**d**) Design diagram of the dimensions of the array of straight channels and the array of restrictive structures. (**e**) Schematic diagram of the capture mechanism of RBCs in the array of restrictive structures (The red circles indicate RBCs).

To achieve high-throughput, real-time assessment of RBC deformability, we developed an automated microfluidic device composed of a microfluidic chip, a programmable flow control system, and an integrated optical imaging system (Figure 1a,b). The core component—the microfluidic chip—is fabricated using standard soft lithography in polydimethylsiloxane (PDMS) and permanently bonded to a glass substrate. The chip has overall dimensions of 30 mm × 15 mm × 0.002 mm and features one inlet and one outlet; its height parameter was carefully optimized to confine RBCs to a flattened state during flow, thereby minimizing rotational artifacts in imaging (Figure 2a).

A dendritic distribution channel was designed at the inlet to uniformly distribute the fluid into 64 parallel subchannels. The inner walls of the subchannels are lined with cylindrical pillars (top radius: 100 μm, bottom radius: 20 μm) (Figure 2b). After passing through the cylindrical pillar region, the fluid flows into a buffer zone and finally enters an array of straight channels (channel width: 20 μm, spacing: 20 μm), forming an ordered fluid transport path (Figure 2c).

Integrated within the chip are 15,800 staggered restrictive structures, each with a length of 60 μm, width of 40 μm, inlet width of 20 μm, and base width of 2 μm. This base width is narrower than the average diameter of RBCs (typically ~7–8 μm), and this dimensional design ensures that cells must deform to pass through; additionally, the structure can effectively capture flowing RBCs (Figure 2d). The restrictive structures are evenly spaced with a center-to-center distance of 20 μm, which provides sufficient space for observing the deformation and recovery dynamics of individual cells between successive structures. When RBCs enter the array of restrictive structures, subsequent cells automatically bypass the occupied structures and flow into the next available ones, ensuring an uninterrupted capture process (Figure 2e).

The chip is enclosed within a temperature-controlled chamber that maintains physiological conditions at 37 °C. Upstream of the test channels, we integrated on-chip filtration structures to remove cell aggregates or debris and prevent clogging during continuous-flow experiments. Whole blood samples are introduced into the chip through a low-dead-volume inlet connected to a micro-pump (a passive check-valve piezoelectric diaphragm pump). The flow rate is tunable from 0.3 to 7 mL/min. In this study, a flow rate of 0.54 mL/min was used to generate shear stresses sufficient to induce observable deformation while preserving steady laminar flow.

To enable automated large-area imaging, a custom-built modular microscope was integrated into the system architecture (Figure 1b). Mounted on a motorized XYZ gantry, this microscope traverses the microfluidic chip along a predefined scanning path, capturing adjacent fields of view that each encompass multiple micro-constriction units. These acquired images are then computationally stitched into a single high-resolution composite image covering the entire chip, enabling global observation of a large population of RBCs rather than being limited to a single region. By coordinating image acquisition with pressure-driven flow control, the platform enables fully automated, high-throughput measurements of RBC deformability at single-cell resolution, eliminating the need for manual alignment or intervention. The device supports the replacement of disposable chips, and its design ensures consistent mechanical conditions across all channels and micro-constrictions—laying a foundation for accurate and scalable characterization of RBC deformability in both research and clinical settings.

### 3.2. Flow Behavior and Shear Distribution

**Figure 3:**
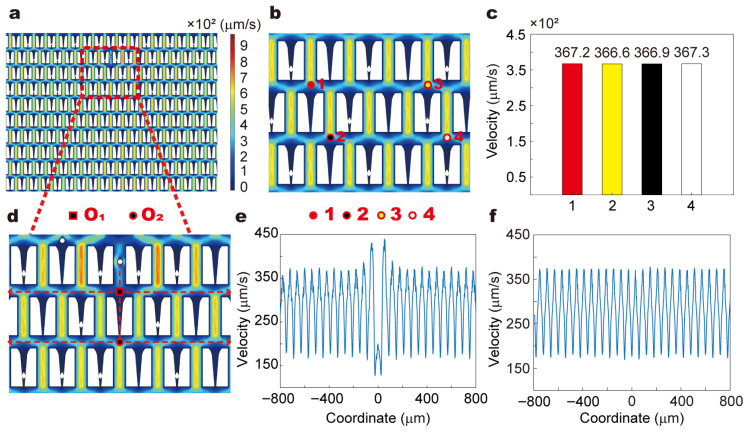
Simulating the Flow Field in Microfluidic Chips. (**a**) Flow field distribution in the microfluidic chip. (**b**) Flow field distribution in microfluidic chips with RBCs captured by restricted structures (above (1) and (2) of the restrictive structure without captured RBCs, and above (3) and (4) of the restrictive structure with captured RBCs). (**c**) Velocity in microfluidic Chips. (**d**) Flow field distribution of RBC entering the bypass channel. (**e**) Velocity distribution in the first row of microstructures downstream of the bypass channel (with O_1_ in panel (**d**) as the origin). (**f**) Velocity distribution in the second row of microstructures downstream of the bypass channel (with O_2_ in panel d as the origin).

**Figure 4:**
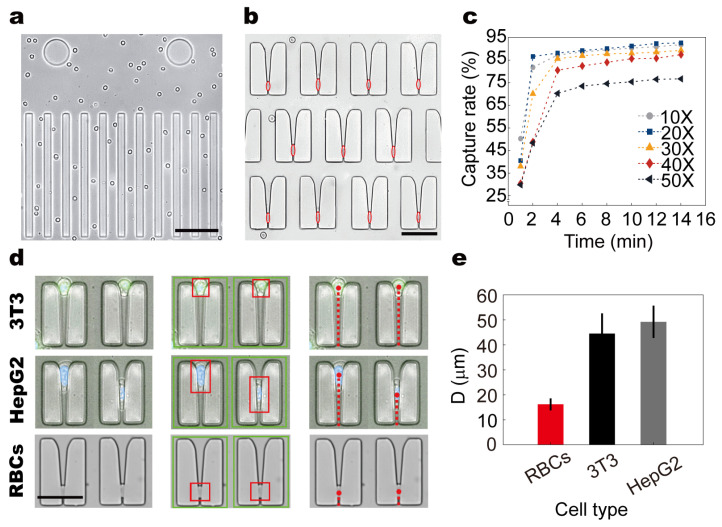
Validation of the behavior of cells in the microfluidic chip. (**a**) Flow images of RBCs in the channel under a 200× microscope, showing that RBCs flow in a flat and orderly manner (Scale bar: 70 μm). (**b**) Image of RBCs captured in the restrictive structures, with the capture locations marked by red hollow ellipses (Scale bar: 40 μm; The red circles indicate RBCs). (**c**) Relationship between cell throughput time and capture efficiency at different dilution ratios (10×, 20×, 30×, 40×, 50×). (**d**) Flowchart of the automated analysis program (Scale bar: 40 μm). (**e**) Bar graph depicting the mean D values of RBCs, HepG2 cells, and 3T3 cells in restrictive structures.

To ensure the stability and uniformity of the flow field in the microfluidic detection system, we performed numerical simulations using COMSOL Multiphysics^®^ 5.3 to investigate whether the presence or movement of RBCs disturbs the local hydrodynamic environment (Figure 3, Supporting Information Text). In the study, RBCs exhibited two typical behaviors: being trapped in restrictive microstructures (Figure 3b) and moving through bypass channels (Figure 3d and Figure S1). Our experiments revealed that when RBCs are trapped in restrictive microstructures, their shape remains unchanged and can be considered as “local obstacles” (Figure 4). In contrast, when RBCs are in bypass channels, they move with the fluid while maintaining their shape (nearly circular) (Video S1). This indicates that when RBCs are trapped in restrictive microstructures, they hardly interact with the flow. When RBCs are in bypass channels, they move with the fluid, but the fluid has minimal influence on their shape.

In this study, a two-dimensional model was constructed based on the microfluidic chip used in the experiments. To simulate the RBCs within the restrictive structures, entities with the same morphology as the RBCs observed in the experiments were embedded in these structures (Figure 3b). On this basis, the impact of cell capture on the fluid flow distribution was simulated. Four points were selected for the analysis of fluid flow changes: above (1) and (2) of the restrictive structure without captured RBCs, and above (3) and (4) of the restrictive structure with captured RBCs (Figure 3b). The results revealed that the flow velocities at these four points were nearly identical, indicating that the capture of cells by the restrictive structures does not influence the fluid flow within the channel (Figure 3c).

We further constructed the process of RBCs flowing in the bypass channels and simulated the impact of RBCs movement on the fluid flow within these channels (Figure 3d and Figure S1). Throughout the entire flow process, the geometric shape of the RBCs remained circular, which is consistent with the experimental observations (Video S1). We analyzed the influence of RBCs at different positions on the fluid flow (Figure 3d and Figure S1), specifically focusing on the effect of RBCs movement on the fluid flow in the lateral channels along their path (Figure 3d). Centered on the vertical direction of RBCs movement, we examined the flow field changes within an 800 μm range to the left and right of the center point (O_1_, O_2_) (Figure 3e,f and Figure S2). The results indicated that the movement of RBCs only caused slight disturbances near the side walls of the bypass channels, with minimal effects on the velocity distribution in other regions.

In conclusion, numerical simulation results demonstrate that whether RBCs are trapped in restrictive microstructures or flow through bypass channels, the disturbances to the surrounding flow field are minimal. The entire microchannel maintains a stable hydrodynamic environment during operation, thereby validating the reliability of deformability measurement based on the trapping positions of RBCs.

While these simulations provide valuable insights into the flow behavior within the microfluidic device, it is important to acknowledge that the current study primarily focuses on the quasi-static behavior of RBCs. Although this approach is useful for understanding the general flow patterns and the impact of RBCs on the hydrodynamic environment, it does not fully capture the dynamic processes and the intricate interactions between RBCs and the surrounding flow. Future work will aim to address these limitations by incorporating more advanced dynamic modeling techniques that can better account for the transient behaviors and fluid-cell interactions, thereby enhancing the comprehensiveness and accuracy of our simulations.

### 3.3. Cell Capture Dynamics and Quantitative Positional Analysis

To validate the cell behavior within the microfluidic chip under steady laminar flow, we conducted systematic observations of RBCs using the integrated modular imaging system. As shown in (Figure 4a), RBCs exhibited a highly ordered, flattened flow pattern along the main channels, maintaining a consistent orientation aligned with the imaging plane. Upon entering the microconstriction array, cells were reliably arrested within restrictive structures, with captured positions indicated by red hollow ellipses (Figure 4b).

To evaluate capture performance under different sample concentrations, whole blood was diluted to 10×, 20×, 30×, 40×, and 50× using PBS and injected into the chip at a constant flow rate of 0.54 mL/min for 14 min. Capture efficiency was quantified as a function of time (Figure 4c). Across all groups, efficiency increased with time and displayed a clear inflection point beyond which the capture rate plateaued. The 10× and 20× groups reached their inflection point within 2 min, while higher dilutions (30×–50×) showed delayed inflection at approximately 4 min. The 20× group achieved 86.6% efficiency at 2 min and peaked at 92.6% after 14 min, indicating that moderate dilution balances throughput and capture performance by reducing cell–cell interference while maintaining sufficient concentration.

To assess the chip’s capacity for biomechanical discrimination, we compared the trapping behavior of RBCs, HepG2 cells, and 3T3 fibroblasts within the constriction array. A custom-developed automated analysis pipeline was employed to extract cell contours, identify capture locations, and compute positional metrics from tiled images (Figure 4d). The key measurement parameter D was defined as the vertical distance (μm) from the centroid of each arrested cell to the base of the microconstriction. Statistical analysis revealed distinct mean D values among the three cell types: RBCs exhibited the deepest capture positions (16.18 ± 2.39 μm), followed by HepG2 cells (44.52 ± 8.08 μm), while 3T3 cells were arrested at the shallowest locations (49.22 ± 6.47 μm) (Figure 4e).

These results confirm that the capture depth is influenced by intrinsic cellular properties—particularly size and deformability. RBCs, owing to their small diameter and high flexibility, penetrate deeper into the constriction before arrest. In contrast, stiffer and larger cells such as 3T3 fibroblasts encounter earlier mechanical resistance and are halted closer to the entrance. This positional segregation enables the chip to function as a high-throughput biomechanical filter, with potential utility for deformability-based diagnostics. Together, these findings demonstrate that the device achieves high-efficiency capture and positional differentiation of various cell types under reproducible flow conditions. Based on these optimized settings (20× dilution, 12–14 min duration), we next investigated how pathophysiological factors, i.e., glucose exposure, alter RBC deformability and mechanical phenotype.

### 3.4. Glucose-Induced Modulation of RBC Deformability

To further investigate how metabolic conditions influence RBC mechanical behavior, we explored the impact of hyperglycemia on cell deformability and morphology using our microfluidic chip system. Building upon the findings from static glucose incubation, we incorporated dynamic in vitro circulation, longitudinal human sampling, and oral glucose tolerance tests (OGTTs) to comprehensively assess the sensitivity and stability of the platform under physiologically relevant glycemic fluctuations.

**Figure 5:**
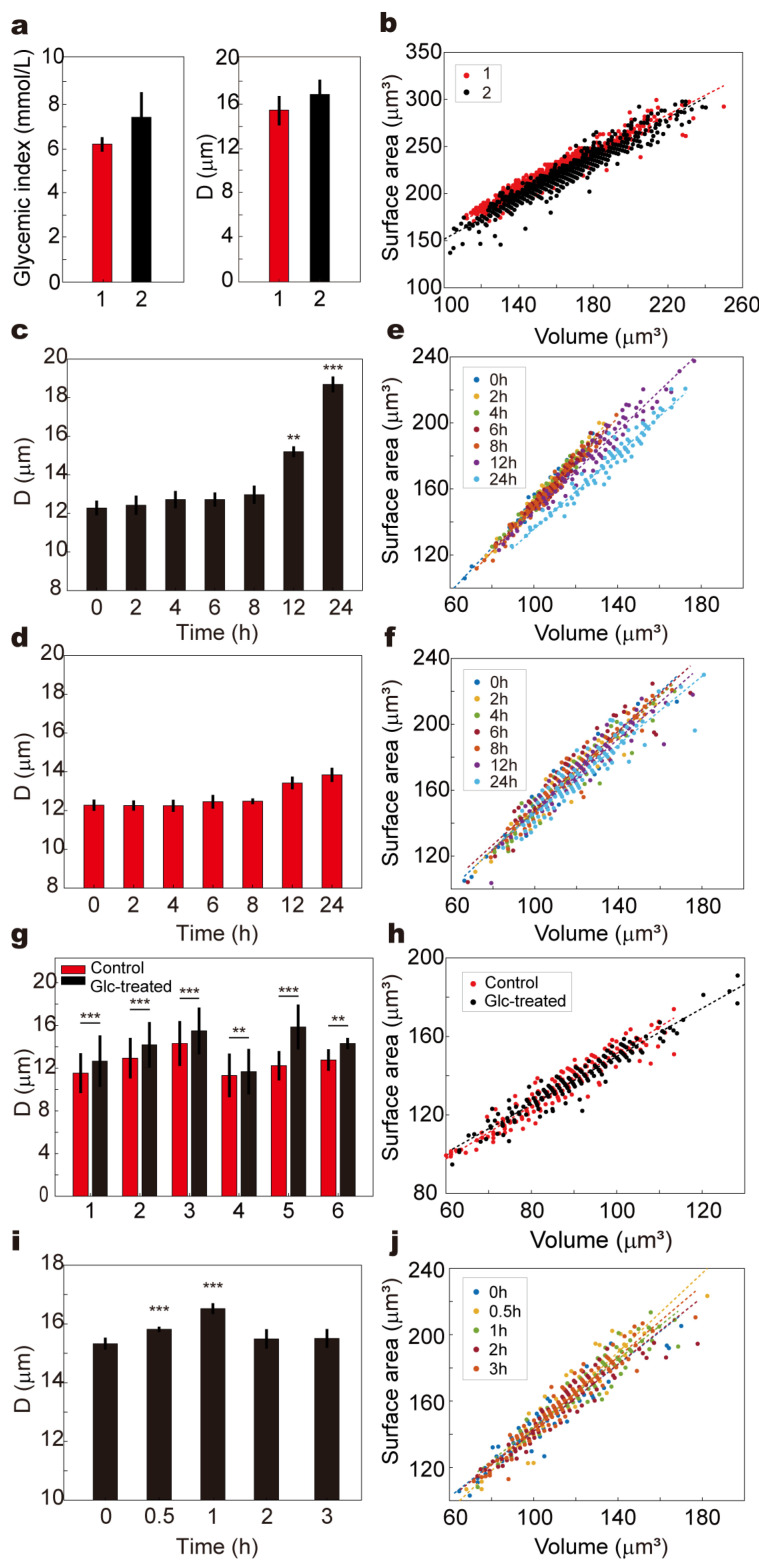
Effects of hyperglycemia on RBC deformability and morphology. (**a**) The variation in blood glucose levels and D values (*p* < 0.01) for two healthy volunteers during a 17-day longitudinal analysis (sampled 2 h postprandially, *n* = 17). (**b**) Scatter plot of the relationship between RBC surface area (S) and volume (V) for two healthy volunteers (*n* = 1000). (**c**) The variation in D values over time under in vitro high-glucose dynamic circulation conditions, with significant differences compared to the static group after 12 h (*p* < 0.01) and at 24 h (*p* < 0.001; *n* = 100). (**d**) The variation in D values over time under in vitro high-glucose static incubation conditions (*n* = 100). (**e**) Scatter plot showing the variation in the relationship between RBC surface area (S) and volume (V). over time in the dynamic circulation experiment (*n* = 100). (**f**) Scatter plot showing the variation in the relationship between RBC surface area (S) and volume (V). over time in the static incubation experiment (*n* = 100). (**g**) The variation in RBC deformability (D values) in whole blood samples from six healthy volunteers after 24 h incubation with high glucose (20 mmol/L), significantly elevated compared to the control group (PBS) (*p* < 0.05; *n* = 200). (**h**) Representative scatter plot of the variation in the relationship between RBC surface area (S) and volume (V) under high-glucose conditions in blood samples from six healthy volunteers (*n* = 200, representative data from one volunteer shown). (**i**) The variation in D values measured using the microfluidic chip at 0, 0.5, 1, 2, and 3 h after glucose ingestion during an OGTT (*n* = 100). (**j**) Scatter plot of the variation in the relationship between RBC surface area (S) and volume (V) over time during an OGTT (*n* = 100). (** *p* < 0.01, *** *p* < 0.001).

We first conducted a 17-day longitudinal analysis using fingertip blood samples from two healthy volunteers. Blood samples were collected 2 h postprandially each day, with one drop analyzed on the microfluidic chip for deformability (D value), and a second drop assessed using a commercial glucose meter. The results revealed a positive association between blood glucose levels and D values: Volunteer 1 exhibited a mean glucose of 5.9 ± 0.68 mmol/L with an average D value of 15.2 ± 1.32 μm, while Volunteer 2 showed significantly higher glucose (8.3 ± 2.28 mmol/L) and D values (16.7 ± 1.32 μm), suggesting reduced deformability under moderate hyperglycemic stress. Corresponding scatter plots of RBC surface area (S) versus volume (V) demonstrated that Volunteer 2’s RBCs shifted toward larger S–V values, consistent with potential cellular swelling or membrane remodeling (Figure 5a,b).

To simulate prolonged exposure to high glucose in vitro, venous blood from a healthy volunteer was supplemented with 20 mmol/L glucose and incubated under two conditions: (1) dynamic circulation through a 2.5 mm inner-diameter tube at 30 mL/min using an infusion pump, and (2) static incubation under identical temperature and gas conditions without flow (Figure 5c,d, Video S2). Samples were collected at 0, 2, 4, 6, 8, 12, and 24 h. In both groups, D values increased over time, but dynamic circulation led to a significantly greater elevation beyond 12 h (*p* < 0.01), reaching maximal divergence at 24 h (*p* < 0.001). This suggests that shear stress potentiates the glycemic impact, likely by enhancing membrane–glucose interactions or promoting cytoskeletal remodeling. The S–V distribution in the dynamic group also exhibited a progressively dispersed trend toward larger values, while the static group displayed a milder shift with aggregation in higher volume regions (Figure 3e,f). To confirm the generalizability of these observations, similar experiments were performed using whole blood from six healthy volunteers. For each sample, one aliquot was incubated in PBS and the other in 20 mmol/L glucose for 24 h at 37 °C. All high-glucose-treated samples exhibited significantly elevated D values compared to controls (*p* < 0.05), and the corresponding S–V distributions shifted to broader and higher-value regions, indicating universal impairment of RBC deformability under hyperglycemic stress (Figure 5g,h and Figure S3).

Additionally, an OGTT was conducted in a healthy volunteer to assess the chip’s ability to track real-time deformability changes during acute glycemic fluctuation. Blood samples collected at 0, 0.5, 1, 2, and 3 h post-glucose ingestion showed that D values significantly increased at 0.5 and peaked at 1 h (*p* < 0.001), returning to baseline by 2 h (Figure 5i). This temporal deformability pattern closely mirrored the blood glucose curve, and the S–V distribution at 1 h demonstrated a marked shift toward larger cell volumes, which resolved by 2–3 h (Figure 5j and Figure S4).

These results support the hypothesis that even moderate or transient hyperglycemia may compromise RBC mechanical properties, potentially impact capillary perfusion and contribute to microvascular complications observed in diabetic pathology.

### 3.5. Dynamic Monitoring of Red Blood Cell Deformability in a T2DM Mouse Model

To assess the long-term impact of metabolic disorders on RBC mechanical behavior under physiological conditions, we established a type 2 diabetes mellitus (T2DM) mouse model and employed our automated microfluidic system to perform dynamic, high-throughput monitoring of RBC deformability over the course of disease progression.

**Figure 6:**
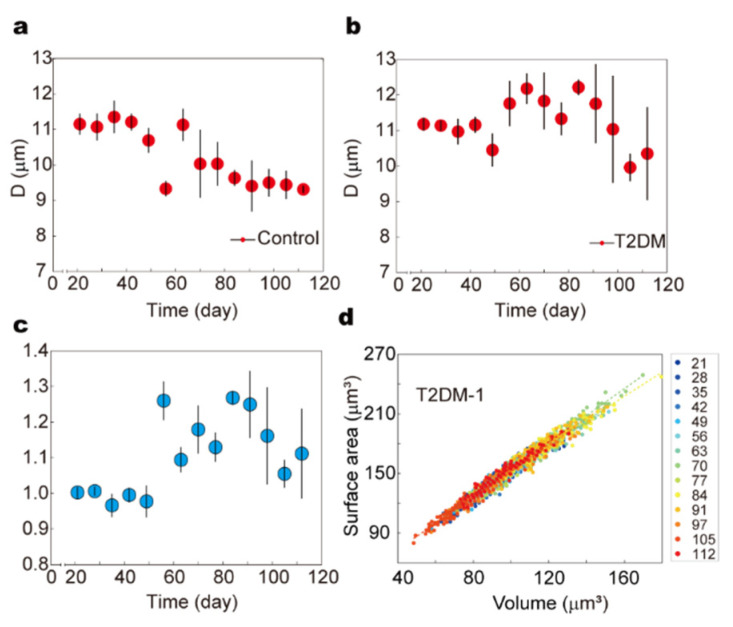
RBC deformation in a T2DM Mouse Model. (**a**) The variation in D-values over time in control group mice (*n* = 100). (**b**) The variation in D-values over time in T2DM mice (*n* = 100). (**c**) The variation in D-norm over time. (**d**) The variation in the relationship between mouse RBC surface area (S) and volume (V) over time (*n* = 100, illustrated with data from one representative T2DM mouse).

Male C57BL/6J mice (3–4 weeks old, SPF-grade) were randomly divided into control and experimental groups. The experimental group received a high-fat diet (60% fat, 20% protein, 20% carbohydrate) for four weeks to induce insulin resistance, followed by intraperitoneal injections of streptozotocin (STZ, 40 mg/kg/day) for five consecutive days to impair pancreatic β-cell function. The control group was maintained on a standard chow diet throughout the experiment. Blood samples were collected weekly over a 112-day period, and fasting blood glucose (FBG) levels were monitored using a glucometer. Concurrently, RBC deformability was assessed using the microfluidic constriction array platform, enabling automated tracking of arrest positions and morphological profiles in a high-throughput manner (Figure 6a,b).

To quantify RBC deformability relative to the healthy baseline, we defined a normalized deformability index D-norm, calculated as the ratio of the mean arrest position in the experimental group to that of the corresponding control group at each time point. As shown in (Figure 6c), the FBG levels in the T2DM group exhibited a gradual and sustained rise, exceeding 11.1 mmol/L by day 56, confirming successful disease induction (Figure S5). Correspondingly, the normalized deformability index D-norm began to increase significantly after day 49, reaching 1.26 ± 0.054, and remained above 1.0 for the remainder of the study, suggesting a persistent decline in RBC deformability under chronic hyperglycemia.

Morphological analysis further revealed progressive alterations in RBC biophysical properties. The distribution of RBC surface area and volume remained compact and stable in the control group throughout the experiment. In contrast, RBCs from the T2DM group exhibited a gradual shift toward higher surface area and volume values, along with increased distributional dispersion over time (Figure 6d and Figure S6). Notably, these changes became pronounced following the onset of sustained hyperglycemia, indicating a correlation between metabolic stress and RBC biophysical remodeling.

The observed increase in RBC volume with comparatively less expansion in surface area suggests a reduction in the surface-area-to-volume (S/V) ratio, which is known to impair cellular deformability by increasing membrane tension and reducing the capacity for elastic deformation. These mechanical alterations may arise from oxidative stress-induced lipid peroxidation, non-enzymatic glycation of membrane proteins, and cytoskeletal disruption—pathological processes previously reported to compromise membrane fluidity and structural integrity in diabetic states.

Our longitudinal data thus provide real-time evidence that hyperglycemia induces progressive mechanical degradation of RBCs. These findings are consistent with previous studies, such as Jin et al., which reported a correlation between elevated HbA1c levels and increased RBC membrane stiffness in T2DM patients due to glycation-induced cytoskeletal crosslinking [67]. Similarly, Lee et al. demonstrated that oxidative stress under hyperglycemic conditions compromises RBC deformability, contributing to impaired microvascular perfusion [68]. Computational models by Deng et al. also support these observations, showing that altered RBC mechanics can exacerbate vascular occlusion in diabetes [69].

In summary, our results underscore the utility of RBC deformability—quantified by microfluidic arrest position and morphological profile—as a sensitive functional biomarker for T2DM progression. The consistent rise in normalized D values and the shift in S–V distributions observed in this study reveal a chronic decline in RBC mechanical adaptability under diabetic conditions, which may contribute to downstream complications such as microvascular occlusion and tissue hypoxia.

## 4. Discussion

This study provides a comprehensive analysis of RBC deformability under hyperglycemic conditions across multiple biological systems—including in vitro glucose exposure, an in vivo T2DM mouse model, real-time monitoring during human OGTTs, and cross-sectional analysis of diabetic patients with varying HbA1c levels. Using our high-throughput microfluidic platform, we demonstrate that both acute and chronic hyperglycemia are associated with a significant reduction in RBC deformability, and these changes can be quantitatively tracked through the D value metric.

Our in vitro experiments revealed that short-term exposure to elevated glucose levels (20 mM) for 24 h significantly reduced RBC deformability. This was evidenced by a stepwise increase in the arrest position (D value), which indicates impaired passage through microchannel constrictions. Mechanistically, this may be attributed to early-stage glycation of membrane proteins and alterations in cytoskeletal structure, which compromise membrane flexibility and enhance cellular stiffness.

In the longitudinal mouse model of T2DM, the normalized D factor increased significantly over time, particularly after the onset of hyperglycemia (fasting blood glucose > 11.1 mmol/L). Additionally, we observed a morphological shift in RBC populations toward higher volume and surface area, suggesting osmotically driven swelling and structural remodeling. Such changes are consistent with previous findings that chronic hyperglycemia impairs RBC ion homeostasis and induces membrane damage via oxidative stress and advanced glycation end-products (AGEs) formation.

In healthy human subjects undergoing OGTTs, we captured a transient but measurable increase in the D value peaking 2 h post-glucose intake, before returning to baseline. This dynamic modulation in deformability in response to fluctuating glucose levels implies that RBCs can acutely respond to metabolic stress even in non-pathological states. These results reinforce the sensitivity of our microfluidic platform to detect real-time mechanical responses under physiological flow conditions.

To extend these findings into the clinical setting, we analyzed peripheral blood samples from a cohort of diabetic patients with varied glycemic control, as reflected by their hemoglobin A1c (HbA1c) levels. As shown in (Figure S7), there was a clear trend: patients with higher HbA1c values generally exhibited higher D values, indicating a decline in deformability with worsening glycemic control. While the correlation is not perfectly linear due to inter-individual variability, the overall distribution supports the hypothesis that cumulative glucose exposure—as indexed by HbA1c—is associated with long-term mechanical deterioration of RBCs. This trend aligns with previous clinical studies reporting increased RBC rigidity in diabetic patients, likely driven by chronic membrane glycation and lipid peroxidation.

Taken together, our data suggest a unified mechanistic framework: glucose exposure, whether transient or sustained, induces biochemical and biophysical changes in RBCs that reduce their ability to deform. This effect can be captured and quantified in a continuous, high-throughput manner using our microfluidic system. The integration of arrest position (D value) and morphological metrics (surface area-to-volume ratio) offers a robust biophysical fingerprint of RBC status under metabolic stress.

Our platform thus serves as a powerful tool to track disease progression and potentially guide therapeutic interventions. The consistency of results across in vitro research, in vivo research, OGTTs, and cross-sectional clinical studies underscores the translational relevance of RBC deformability as a biomechanical biomarker of glycemic burden. In particular, the progressive increase in D values as a function of HbA1c across patient samples suggests that RBC mechanical testing may serve as a functional supplement to traditional biochemical markers, particularly in evaluating microvascular risk in diabetic populations.

In future work, it will be valuable to expand the clinical cohort to investigate the influence of disease duration, age, treatment regimen, and comorbidities on RBC mechanical properties. Moreover, integrating biochemical assays of membrane protein glycation and lipid oxidation could further elucidate the molecular underpinnings of the observed mechanical decline. Nonetheless, this study lays the foundation for RBC biomechanics to be incorporated into diagnostic and prognostic frameworks for diabetes and other systemic conditions affecting microcirculation.

## 5. Conclusions

In this study, we developed a fully automated, high-throughput microfluidic imaging system capable of quantifying RBC deformability across thousands of individual cells under physiologically relevant flow conditions. By integrating modular microscopy and position-resolved microconstriction arrays, we achieved large-area imaging and precise measurement of cellular arrest positions as a deformability index (D value). Applying this platform across multiple experimental contexts—including in vitro glucose exposure, a T2DM mouse model, OGTTs in healthy volunteers, and clinical samples from diabetic patients—we consistently observed that elevated blood glucose levels and metabolic stress correlate with decreased RBC deformability, reflected by a significant increase in D values and altered cell morphology. These results reveal that hyperglycemia induces biomechanical changes in RBCs prior to irreversible damage, likely due to membrane stiffening from oxidative stress and glycation. Our findings highlight RBC deformability as a sensitive, quantifiable biomechanical marker for glycemic stress and diabetes progression, offering new potential for early diagnosis, disease monitoring, and personalized metabolic health assessment.

## Data Availability

The original contributions presented in this study are included in the article/Supplementary Materials. Further inquiries can be directed to the corresponding author.

## Supplementary Materials

The following supporting information can be downloaded at: https://www.mdpi.com/article/10.3390/bios15110758/s1, Figure S1: Simulation of the local flow field during RBC movement in the bypass channel. (the RBC is marked with a red dashed circle) (a) The RBC flows downward and locates in the left-biased flow channel of the longitudinal bypass channel outlet region. (b) The RBC flows to the left and downward into the transverse bypass channel. (c) The RBC flows downward and stays in the middle-front section of the longitudinal bypass channel. (d) The RBC flows downward and locates in the terminal section of the longitudinal bypass channel. (e) The RBC flows to the right into the transverse bypass channel. (f) The RBC flows to the right and downward into the inlet region of the longitudinal bypass channel; Figure S2: Velocity magnitude distribution in the bypass channel. All subfigures consist of two panels (left and right). The left panel shows the velocity magnitude distribution in the first row of microstructures downstream of the bypass channel (with O_1_ in Figure S1 as the origin), and the right panel shows that in the second row of microstructures (with O_2_ in Figure S1 as the origin); Figure S3: Scatter plot of the variation of the relationship between RBC surface area (S) and volume (V) under high glucose conditions in blood samples from six healthy volunteers (*n* = 200); Figure S4: Blood glucose levels of one volunteer during an oral glucose tolerance test (OGTT). Blood glucose concentrations (mmol/L) were measured at 0, 0.5, 1, 2, and 3 h after glucose ingestion; Figure S5: The variation of fasting blood glucose levels over time in control and type 2 diabetes mellitus (T2DM) mice. a. The variation of fasting blood glucose levels over time in control group mice. b. The variation of fasting blood glucose levels over time in type 2 diabetes mellitus (T2DM) mice; Figure S6: Scatter plot of the variation of the relationship between mouse RBC surface area (S) and volume (V) over time (*n* = 200); Figure S7: RBCs deformability in the Development of Diabetes. a. The proportion of Hemoglobin A1c (HbA1c) to total hemoglobin in different patients. b. RBCs deformation (*D* values) in different patients; Video S1: Flow process of RBCs in the bypass channel; Video S2: The blood oscillated through a 2.5 mm diameter tube at a flow rate of 30 mL/min.
