# Supplementary material for: A Microfluidic Device for Detecting the Deformability of Red Blood Cells
Source: Biosensors (Basel). 2025 Nov 14;15(11):758. doi: 10.3390/bios15110758 (PMC12649979; doi:10.3390/bios15110758)
Supplement: Supplementary file 1 [file biosensors-15-00758-s001.zip › biosensors-3914852-Supplementary.pdf]

Supplementary Information

A microfluidic device for detecting the deformability of red blood cells

Wenyan Liu[1,2,†], Liqiang Xie1[,†], Jiangcun Yang[3], Xiaobo Gong [4], Dan Sun[1,*], Ce Zhang[1,*]

[1] *State Key Laboratory of Photon-Technology in Western China Energy, Institute of Photonics and Photon-Technology, Northwest University, No. 1, Xuefu Avenue, Xi'an, 710127, China*

[2] *School of Physics, Northwest University, No. 1, Xuefu Avenue, Xi'an, 710127, China*

[3] *Department of Transfusion Medicine, Shaanxi Provincial People's Hospital, Xi'an 710068, China*

[4] *School of Ocean and Civil Engineering, Shanghai Jiao Tong University, Shanghai, 200240, China*

[*] *Correspondence: sund@nwu.edu.cn (D.S.); zhangce.univ@gmail.com (C.Z.)*

[†] *These authors contributed equally to this work*

This PDF file includes:

Supplementary Text

Figs. S1 to S7

Other supplementary materials for this manuscript include the following:

Video S1 to S2

Supporting Information Text

1. Computational Tools Employed

The computational tool employed in this study is COMSOL Multiphysics® 5.3, a widely recognized commercial numerical simulation software that has been extensively used in both academic and industrial research, particularly in the fields of microfluidics and biofluids [1-5]. In the study, we established a two-dimensional geometric model based on the microfluidic chip used in the experiments and simulated the "laminar flow" within the chip using this software [6-9].

2. Governing Equations

The flow field calculations strictly adhere to the classical governing equations for incompressible fluids, which are widely adopted in microfluidic simulations [10-11]:

Continuity Equation:

$$\frac{\partial u_x}{\partial x} + \frac{\partial u_y}{\partial y} = 0$$

Derived from the law of mass conservation, this equation ensures that mass is neither created nor destroyed during fluid flow. It is a fundamental constraint for incompressible flow and has been extensively used in microfluidic studies to validate flow field stability, especially in low-Reynolds-number scenarios [12].

Navier-Stokes Equations:

x-component:

$$\rho(\frac{\partial u_x}{\partial t} + u_x\frac{\partial u_x}{\partial x} + u_y\frac{\partial u_x}{\partial y}) = -\frac{\partial p}{\partial x} + \mu(\frac{\partial^2 u_x}{\partial x^2} + \frac{\partial^2 u_x}{\partial y^2})$$

y-component:

$$\rho(\frac{\partial u_y}{\partial t} + u_x\frac{\partial u_y}{\partial x} + u_y\frac{\partial u_y}{\partial y}) = -\frac{\partial p}{\partial y} + \mu(\frac{\partial^2 u_y}{\partial x^2} + \frac{\partial^2 u_y}{\partial y^2})$$

This equation describes momentum conservation in fluids and is the core theoretical basis for simulating laminar flow in microchannels. For low-Reynolds-number flows (Re ≈ 0.1) in microfluidic chips, the inertial terms are negligible, and the equation simplifies to a balance between pressure gradient and viscous forces. Here, ρ (≈1000 kg/m^3) is the fluid density (matching physiological buffer), μ (≈1×10^{-3} Pa·s) is dynamic viscosity, p is pressure, and u_x, u_y are velocity components.

3. Boundary Conditions

The boundary conditions were set according to the design of the microfluidic chip used in the experiment, the actual parameters employed in the experiment, and the basic principles of fluid mechanics, as follows:

Inlet Boundary: A constant flow velocity boundary was applied, with the velocity set to 3 mm/s. This setup simulates the scenario in microfluidic experiments where a micropump provides stable fluid driving, ensuring the fluid enters the chip channel at a controllable and steady velocity, consistent with experimental operation logic.

Outlet Boundary: A zero-gauge pressure boundary (gauge pressure = 0 Pa) was adopted.

This configuration effectively simulates the real discharge state of the chip's open flow channel, preventing unreasonable backflow at the outlet and ensuring the overall stability of the flow field, which aligns with the actual working environment of microfluidic chips.

Solid Wall Boundaries: All solid walls—including the walls of restrictive microstructures, channel sidewalls, and the surfaces of the embedded fixed RBC geometries—were set to no-slip boundaries $(u_x = 0, u_y = 0)$. This setup follows the basic fluid mechanics principle that "fluid velocity at solid walls is zero," accurately reflecting the interaction between the fluid and solid interfaces.

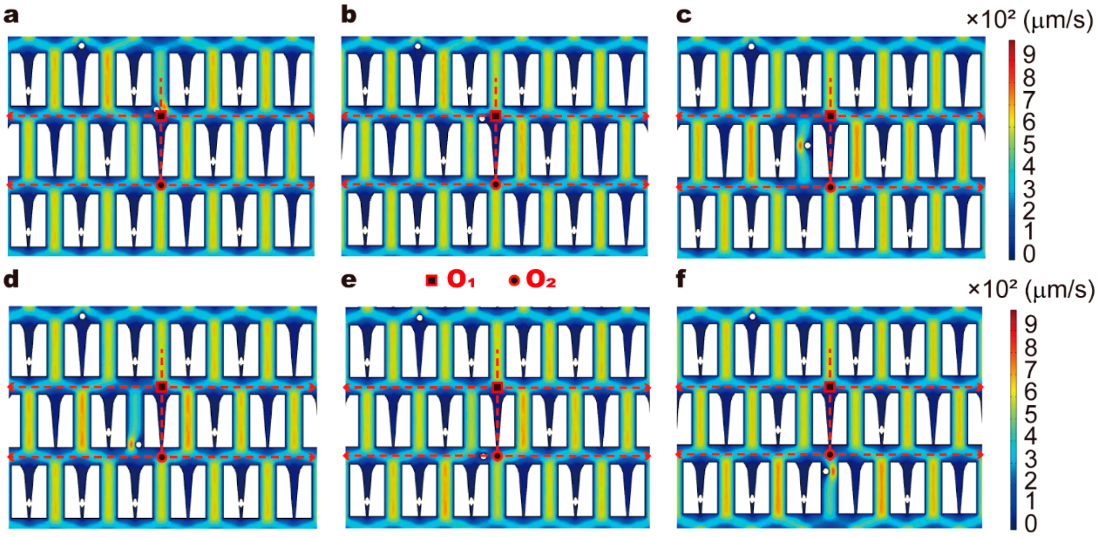

Fig. S1: Simulation of the local flow field during RBC movement in the bypass channel. (the RBC is marked with a red dashed circle) (a) The RBC flows downward and locates in the left-biased flow channel of the longitudinal bypass channel outlet region. (b) The RBC flows to the left and downward into the transverse bypass channel. (c) The RBC flows downward and stays in the middle-front section of the longitudinal bypass channel. (d) The RBC flows downward and locates in the terminal section of the longitudinal bypass channel. (e) The RBC flows to the right into the transverse bypass channel. (f) The RBC flows to the right and downward into the inlet region of the longitudinal bypass channel.

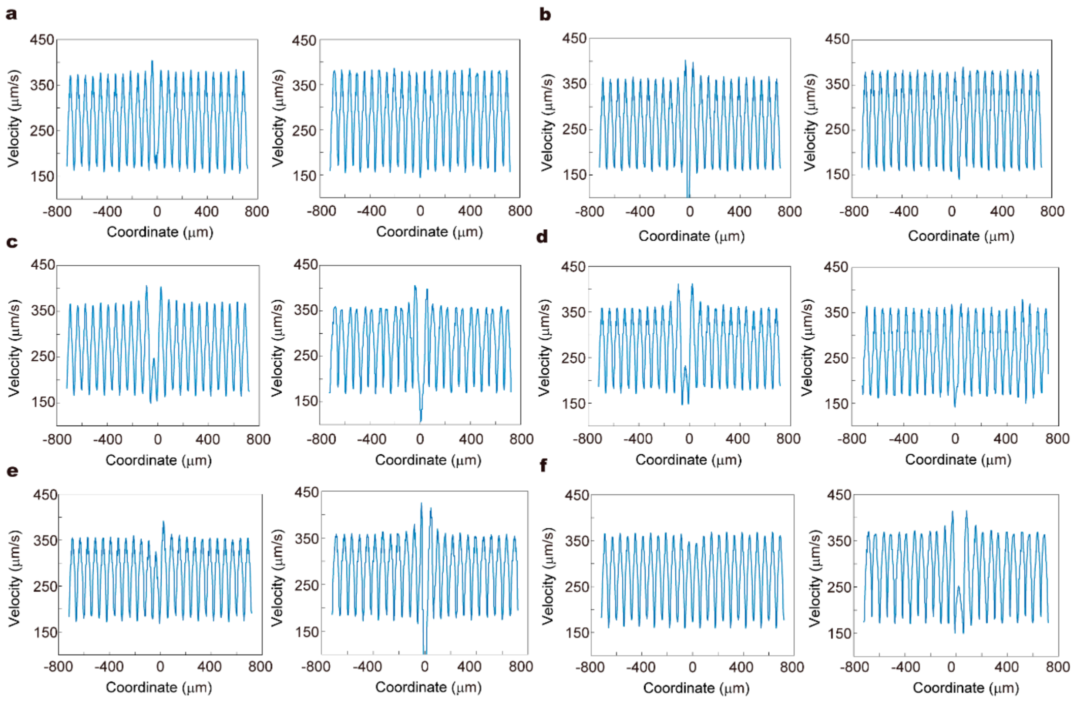

Fig. S2: velocity magnitude distribution in the bypass channel. All subfigures consist of two panels (left and right). The left panel shows the velocity magnitude distribution in the first row of microstructures downstream of the bypass channel (with O_1 in Fig. S1 as the origin), and the right panel shows that in the second row of microstructures (with O_2 in Fig. S1 as the origin).

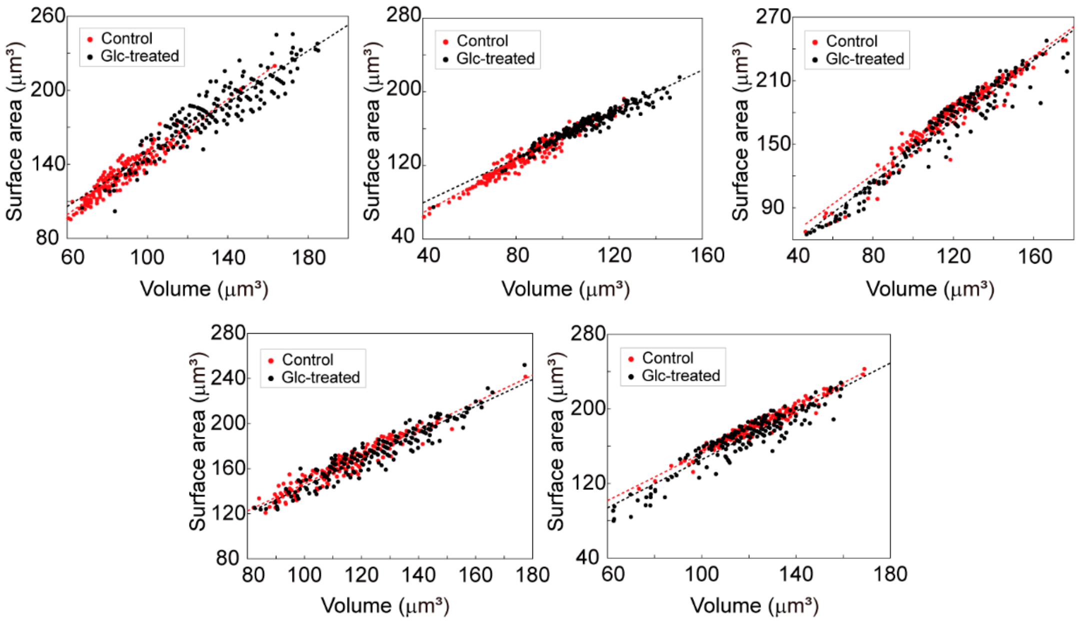

Fig. S3: Scatter plot of the variation of the relationship between RBC surface area (S) and volume (V) under high glucose conditions in blood samples from six healthy volunteers (n = 200).

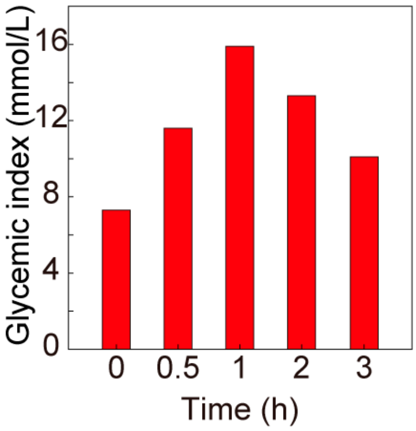

Fig. S4：Blood glucose levels of one volunteer during an oral glucose tolerance test (OGTT). Blood glucose concentrations (mmol/L) were measured at 0, 0.5, 1, 2, and 3 hours after glucose ingestion.

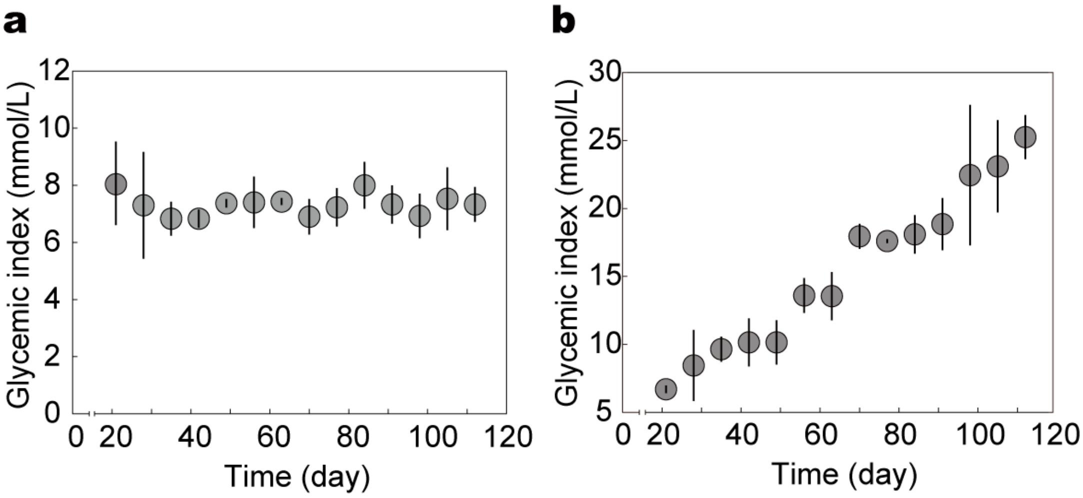

Fig. S5：The variation of fasting blood glucose levels over time in control and type 2 diabetes mellitus (T2DM) mice. a. The variation of fasting blood glucose levels over time in control group mice. b. The variation of fasting blood glucose levels over time in type 2 diabetes mellitus (T2DM) mice.

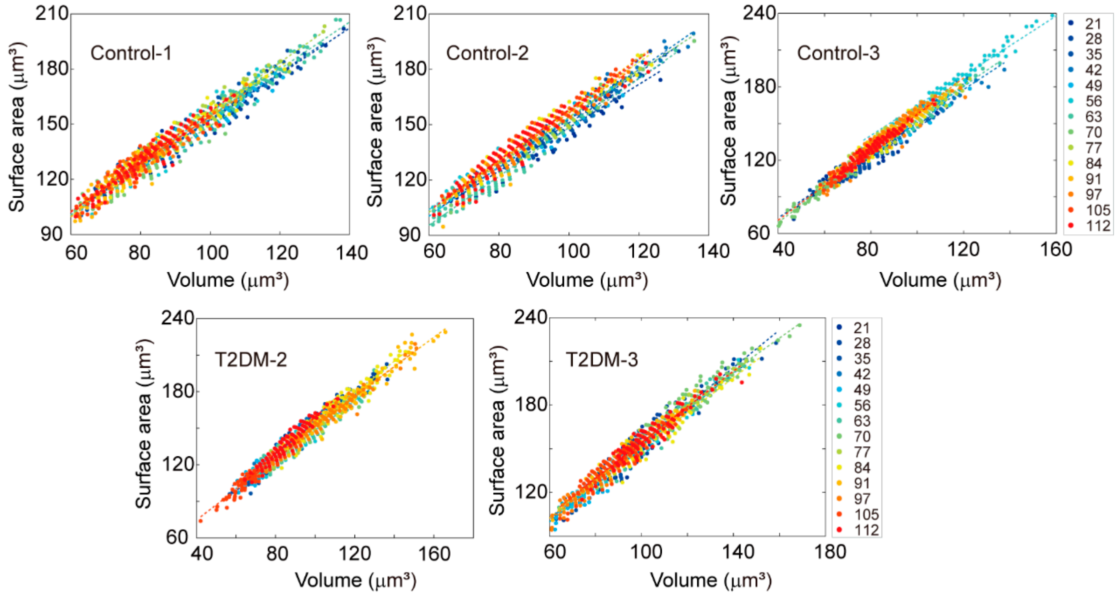

Fig. S6: Scatter plot of the variation of the relationship between mouse RBC surface area (S) and volume (V) over time (n = 200).

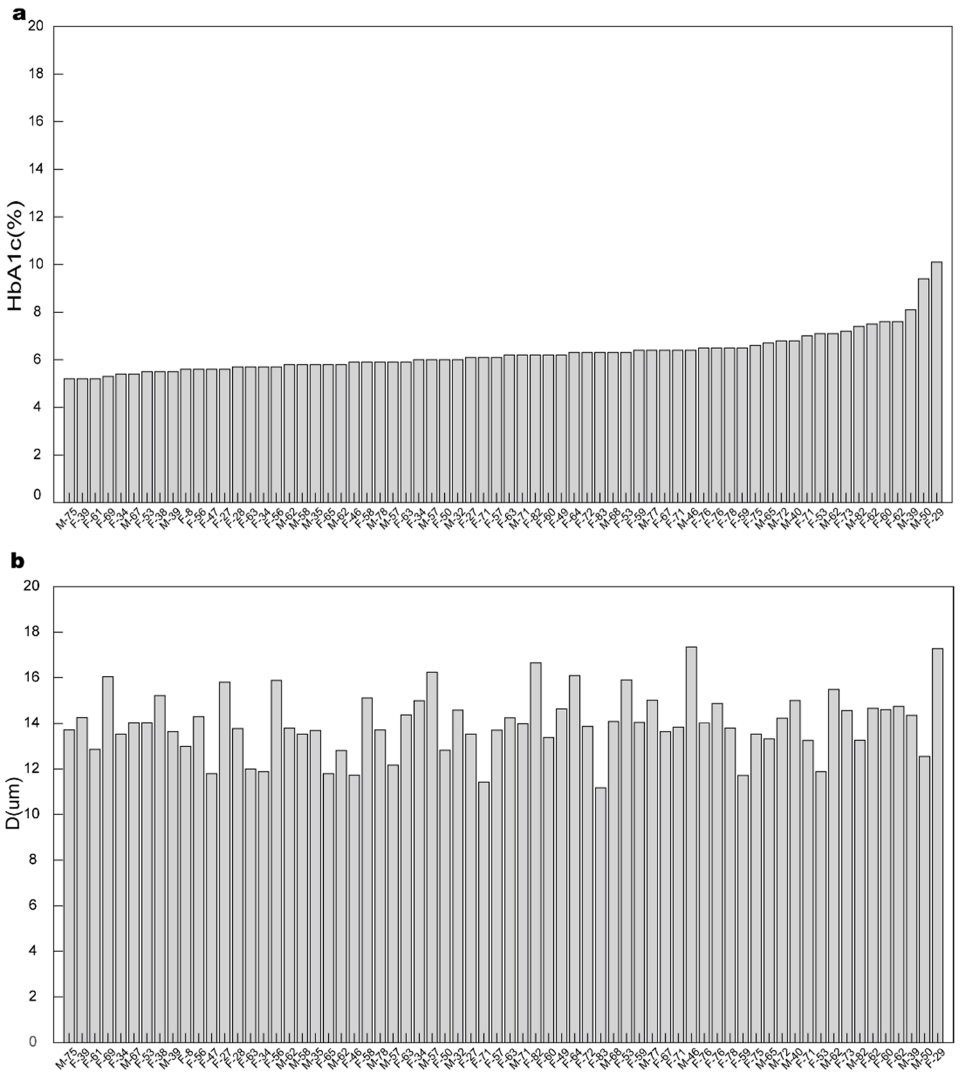

Fig. S7: RBCs deformability in the Development of Diabetes. a. The proportion of Hemoglobin A1c (HbA1c) to total hemoglobin in different patients. b. RBCs deformation (*D* values) in different patients.

Video S1: Flow process of RBCs in the bypass channel.

Video S2: The blood oscillated through a 2.5 mm diameter tube at a flow rate of 30 mL/min.

References

[1] Meng, X.; Yu, Y.; Jin, G. Numerical Simulation and Experimental Verification of Droplet Generation in Microfluidic Digital PCR Chip. *Micromachines* **2021**, *12*, 409.

[2] Somaweera, H.; Haputhanthri, S. O.; Ibraguimov, A.; Pappas, D. On-chip gradient generation in 256 microfluidic cell cultures: simulation and experimental validation. Analyst **2015**, 140, 5029-5038.

[3] Bachal, K.; Yadav, S.; Gandhi, P.; Majumder, A. Design and validation of a flowless gradient generating microfluidic device for high-throughput drug testing. Lab Chip **2023**, 23(2), 261-271.

[4] Liu, G.G.; Huang, H.B.; Chen, Z.S.; Lin, H.X.; Liu, H.; Huang, X.; Guo, W.Z. Design automation for continuous-flow microfluidic biochips: A comprehensive review. Integration **2022**, 82, 48-66.

[5] Islam, M.Z.; Tsui, Y.Y. Quasi-3D Modeling and Efficient Simulation of Laminar Flows in Microfluidic Devices. *Sensors* **2016**, *16*, 1639.

[6] Zahorodny-Burke, M.; Nearingburg, B.; Elias, A.L. Finite element analysis of oxygen transport in microfluidic cell culture devices with varying channel architectures, perfusion rates, and materials. Chemical Engineering Science **2011**, 66(23), 6244-6253.

[7] Meng, X.; Yu, Y.; Jin, G. Numerical Simulation and Experimental Verification of Droplet Generation in Microfluidic Digital PCR Chip. *Micromachines* **2021**, *12*, 409.

[8] Juraeva, M.; Kang, D.-J. Design and Mixing Analysis of a Passive Micromixer with Circulation Promoters. *Micromachines* **2024**, *15*, 831.

[9] Waqas, M.; Janusas, G.; Naginevičius, V.; Palevicius, A. The Design and Investigation of Hybrid a Microfluidic Micromixer. *Appl. Sci.* **2024**, *14*, 5315.

[10] Bird, R. B.; Stewart, W. E.; Lightfoot, E. N. Transport Phenomena, Revised 2nd ed.; John Wiley & Sons: Hoboken, NJ, USA, **2007**.

[11] COMSOL Multiphysics. Microfluidics Module User's Guide; COMSOL AB: Stockholm, Sweden,**2021**.

[12] Low, W.S.; Kadri, N.A.; Abas, W.A. Computational fluid dynamics modelling of microfluidic channel for dielectrophoretic BioMEMS application. ScientificWorldJournal **2014**, 2014, 961301.
